# Information and Communication Technology Use in Suicide Prevention: Scoping Review

**DOI:** 10.2196/25288

**Published:** 2021-05-04

**Authors:** Jessica Rassy, Cécile Bardon, Luc Dargis, Louis-Philippe Côté, Laurent Corthésy-Blondin, Carl-Maria Mörch, Réal Labelle

**Affiliations:** 1 Center for Research and Intervention on Suicide, Ethical Issues and End-of-Life Practices Université du Québec à Montréal Montréal, QC Canada; 2 Research Center Institut universitaire en santé mentale de Montréal Montréal, QC Canada; 3 School of Nursing Université de Sherbrooke Longueuil, QC Canada; 4 Quebec Network on Nursing Intervention Research Montréal, QC Canada; 5 Department of Psychology Université du Québec à Montréal Montréal, QC Canada; 6 Algora Lab Université de Montréal Montréal, QC Canada; 7 Mila, Quebec Artificial Intelligence Institute Montréal, QC Canada; 8 Department of Psychiatry Université de Montréal Montréal, QC Canada

**Keywords:** suicide prevention, information and communication technology, scoping review, mobile phone

## Abstract

**Background:**

The use of information and communication technology (ICT) in suicide prevention has progressed rapidly over the past decade. ICT plays a major role in suicide prevention, but research on best and promising practices has been slow.

**Objective:**

This paper aims to explore the existing literature on ICT use in suicide prevention to answer the following question: what are the best and most promising ICT practices for suicide prevention?

**Methods:**

A scoping search was conducted using the following databases: PubMed, PsycINFO, Sociological Abstracts, and IEEE Xplore. These databases were searched for articles published between January 1, 2013, and December 31, 2018. The five stages of the scoping review process were as follows: identifying research questions; targeting relevant studies; selecting studies; charting data; and collating, summarizing, and reporting the results. The World Health Organization suicide prevention model was used according to the continuum of universal, selective, and indicated prevention.

**Results:**

Of the 3848 studies identified, 115 (2.99%) were selected. Of these, 10 regarded the use of ICT in universal suicide prevention, 53 referred to the use of ICT in selective suicide prevention, and 52 dealt with the use of ICT in indicated suicide prevention.

**Conclusions:**

The use of ICT plays a major role in suicide prevention, and many promising programs were identified through this scoping review. However, large-scale evaluation studies are needed to further examine the effectiveness of these programs and strategies. In addition, safety and ethics protocols for ICT-based interventions are recommended.

## Introduction

### Background

Information and communication technology (ICT) has been used for suicide prevention over the past decade. Moreover, there is a growing body of evidence supporting the use of ICT in the development of promising suicide prevention practices [1,2]. ICT can be used to screen individuals at risk of suicide on the web; offer information and help regarding suicidal thoughts and behavior; and offer web-based assessment, interventions, and follow-up [1-6]. The use of ICT widens accessibility to hard-to-reach individuals who do not always seek help in person and offers treatment opportunities to communities with lower access to care, such as rural communities. The use of ICT in suicide prevention can also help professionals offer better care to their patients by combining multiple approaches, such as using mobile apps to monitor symptoms or providing web-based therapeutic programs [7,8].

Many opportunities arise when using ICT to expand suicide prevention strategies. However, the pace at which ICT is advancing makes it difficult to keep up with, especially from a research perspective. To make better use of these different web-based suicide prevention strategies, a better understanding of these different uses of ICT is necessary. We identified 3 major questions. First, a better understanding of how to emphasize the technical aspects of ICT regarding the development, maintenance, privacy, and life cycle of the technology would help make better choices regarding which ICT to use in what context. For example, machine learning offers many possibilities for identifying at-risk individuals, but major technical and ethical considerations must be described and taken into account. Second, we need to improve our understanding of ICT use in suicide prevention. To support adequate decision making, it is important to know whether ICT-based suicide prevention strategies reach different clienteles or if we are reaching the same individuals differently. Third, a better understanding of the structure and efficacy of various types of current web-based assessments and interventions is also important. For instance, can artificial intelligence (AI) and machine learning be used to optimize and accelerate the assessment of individuals at risk? Is a suicide crisis intervention by chat as effective as talking over the phone or in person? Are web-based cognitive behavioral therapy (CBT) programs as effective for suicide prevention as face-to-face CBT programs? Before shifting toward using and recommending the use of these different ICT-based assessments and interventions, it should be noted that their effectiveness has yet to be demonstrated in the literature.

### Current Study

The available literature often refers to a specific type of ICT as opposed to a general overview of existing ICT-based strategies in mental health and suicide prevention. More precisely, existing literature reviews have focused on specific ICTs such as smartphone tools [1], web-based interventions [3], mobile apps [4], and social media [5] or specific age groups such as youth [6]. Based on this background and a rapidly evolving corpus of research, we set out to review the evidence supporting the use of all types of ICTs in different levels of suicide prevention strategies. More precisely, we carried out a scoping review to identify the best and most promising practices for ICT use at all levels of suicide prevention, which are described by the World Health Organization as the universal (entire population), selective (specific subpopulations), and indicated (high-risk individuals) levels [9]. ICT was defined as all materials, software, or services used to collect, process, and transmit information; this includes electronic, computer, telecommunications, multimedia, and internet technologies [10]. AI was defined as all theories and techniques used to develop machines capable of simulating intelligence [7].

## Methods

### Scoping Review Framework

We used the 5-stage scoping framework developed by Arksey and O’Malley [11] and adapted by Levac et al [12] to review peer-reviewed publications. These stages are (1) identifying research questions; (2) identifying relevant studies; (3) selecting studies; (4) charting data; and (5) collating, summarizing, and reporting results. A scoping review allows researchers to explore the available data on various aspects of a theme instead of just one [11,12]. This provides an overview of the relevant literature that helps to identify the various ICT and suicide prevention strategies examined by researchers since 2013 and to document their effects. The Center for Research and Intervention on Suicide, Ethical Issues, and End-of-Life Practices decided to better understand the use of ICT in suicide prevention as of 2013 following the publication of the book *Suicide prevention and new technologies: Evidence-based practice* [13]. This book provides an overview of new technologies in suicide prevention and demonstrates the need for further research in this area. As ICT tools, contents, and use evolve rapidly, a 5-year timeframe seems relevant to address the current state of ICT preventive practices and research. For example, major social networks have changed their policies toward content related to suicidality. During the 2019 International Suicide Prevention Day, Facebook revised its policy of preventing self-harm and suicide by banning graphic representations of suicidality, which could change the level of exposure of individuals vulnerable to suicide. At the same time, the rapid development of scientific knowledge and technology has been noted many times. For example, a review of the scientific literature published in 2013 identified 5 scientifically evaluated mobile apps for child and adolescent mental health [14], whereas Grist et al [15] identified 19 more. In addition, web-based platforms such as Parler or 4Chan were not as widely known and used 10 years ago. Including older technologies and neglecting to consider the rapid content change of internet content over the years may skew results toward obsolete prevention strategies.

### Summary of Search Strategy

#### Identifying the Research Question

The research question identified for this scoping review aims to guide future program development and implementation by establishing an inventory of strategies using ICT that have been the subject of research in recent years. Following the PRISMA (Preferred Reporting Items for Systematic Reviews and Meta-Analyses) recommendations, the research question was developed using the PICO (population, intervention, comparison, and outcomes) conceptual tool [16]. Note that an adaptation of PICO was made through an amalgamation with the SPIDER (sample, phenomenon of interest, design, evaluation, research type) tool by Methley et al [17] to facilitate the consideration of the psychosocial nature of the research object. Consequently, the research question was “How information and communication technology (ICT) can be used in intervention and prevention of suicide among the general population, people at risk, or suicidal people of all ages and conditions combined to contribute to the reduction of any indicators relating to suicidality?” This question was selected to promote the instrumental use of the research results [18].

#### Identifying Relevant Studies

The search strategy consisted of identifying key concepts related to ICT-based suicide prevention and intervention, developing a provisional syntax specific to each database for pretesting, and determining the definitive syntax based on the pretest results. The keywords used to construct research syntaxes were extracted from natural (everyday vocabulary) and controlled (indexer terms) language terms and based on the central concepts associated with the research questions. The researched concepts were suicidal behavior, ICT (AI, machine learning, application, social network, and mobile technology), intervention (information, education, prevention, decision making, and intervention), and program evaluation. The final syntax based on these keywords and used with each database searched is presented in Multimedia Appendix 1. PubMed, PsycINFO, Sociological Abstracts, and IEEE Xplore were searched for publications from January 1, 2013, to December 31, 2018. The selection of each database was carefully thought out by the research team (2 senior researchers in suicidology, 2 ICT experts, 1 postdoctoral student, 1 doctoral student, and 1 specialized librarian who provided support) to include data in the medical field, psychology, social and behavioral sciences, and ICT. The number of databases chosen was in line with that of other reviews that focused on specific ICTs and mental health [5,19]. In addition, the choice of databases was based on the following 2 criteria: the number of documents they contain and the degree of overlap of the content indexed in each database [20]. The final choice was made to select high quantities of indexed documents in databases and databases with less content overlap to maximize the completeness of the retrieval process.

##### Selecting Studies

**Figure 1:**
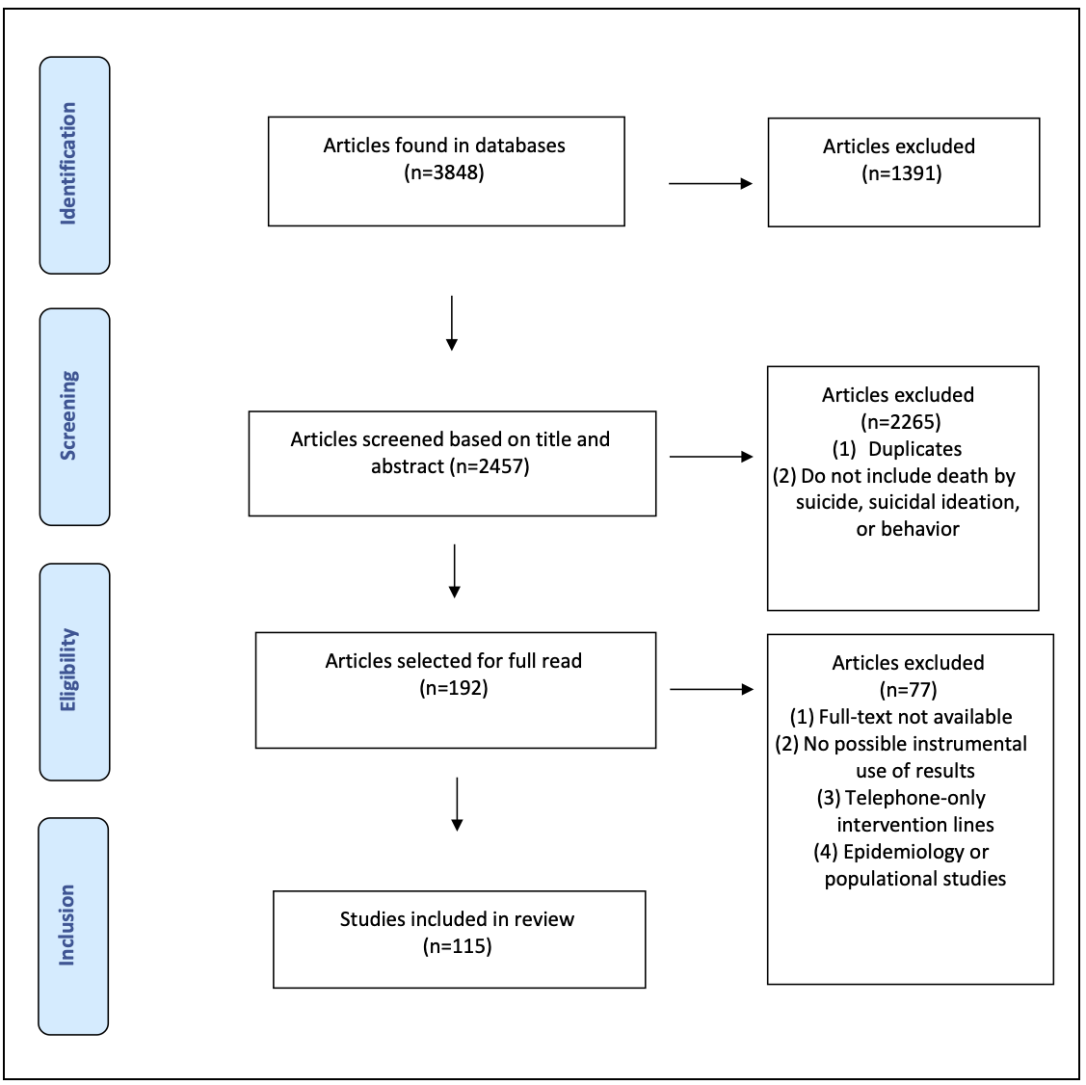
PRISMA (Preferred Reporting Items for Systematic Reviews and Meta-Analyses) flow diagram.

The final search syntax identified 3848 publications that were imported for EndNote selection. From these, 1391 duplicates were removed. The remaining 2457 publications were then sorted according to the inclusion and exclusion criteria applied by 5 research assistants against titles and abstracts only. Interrater reliability was assessed for 100 publications. Agreement reached 89% and a Cohen κ statistic of 0.704 was achieved, which is considered substantial by Cohen [21]. The studies covered in our review used various methods (qualitative, quantitative, and mixed methods) and were drafted in English or French. They had to provide original empirical or descriptive data. Literature reviews, editorials, theoretical articles, and studies focused only on ethical or legal issues surrounding ICT-based suicide prevention were excluded. The included articles had to directly address suicide prevention strategies using ICT. All studies that used death by suicide, suicidal ideation, or suicide behavior as outcomes were considered. The PRISMA flow chart [16] of our study presented in Figure 1 provides an overview of our study selection process.

##### Charting, Collating, Summarizing, and Reporting Data

A form was developed in Microsoft Excel to retrieve pertinent information from selected qualitative, quantitative, and mixed methods studies to optimize data charting. A research team tested the form before use. It included 10 items: title, objectives, research design, instruments of measure, type of suicide prevention (universal, selective, or indicated), technology category, participant characteristics, results associated with effects and benefits regarding lowering suicidal behavior, clinical and scientific contributions, and recommendations. The research team thoroughly examined 5 independent studies with 10 items and then compared the results. Following this analysis, precision was applied to the exclusion criteria of the studies. Verbal telephone interventions were excluded from this review. Considering the large quantity of data available on suicide prevention telephone interventions [22-24], studies addressing these types of interventions were excluded from this scoping review. However, interventions based on text messaging and smartphone apps were included as new ICT-based interventions relevant to this review. After criteria validation, a summary of pertinent information was prepared for each study included. No systematic methodological quality evaluation was carried out in accordance with the scoping review methodology [11].

## Results

### General Information

Our search of PubMed, PsycINFO, Sociological Abstracts, and IEEE Xplore yielded 3848 articles. After removing 1391 duplicates and 2265 articles based on a perusal of title and abstract, 192 texts were read in full for the final screening. This allowed us to remove an additional 77 articles, leaving 115 articles for the scoping review. We also noticed through our analysis that the number of publications increased in 2016 and 2017, and the scientific articles focused more on selective and indicated suicide prevention strategies.

### Classification of Interventions’ Efficacy

In the context of this scoping review, we aim to provide a general portrait of existing suicide prevention strategies based on ICT. It does not aim to determine the quantitative effectiveness of such interventions. Therefore, a prevention strategy is considered effective when it is based on rigorous theory and when it has been evaluated by a minimum of 2 studies with a quasi-experimental approach [25]. Typically, in traditional systematic reviews, identifying *evidence-based practices* requires a weighting of the methodological quality of primary studies, unlike the scoping review [12]. If the relevant studies are randomized trials, the body of evidence begins with high certainty. If the relevant studies are observational, the body of evidence begins with low certainty [26]. Certain strategies may, however, be presented as *promising* when at least one observational or randomized study supports its efficacy. Therefore, we considered all interventions that have demonstrated effectiveness in any type of research design (qualitative, quantitative, or mixed studies).

### Universal Suicide Prevention Strategies

In total, 10 of the studies selected referred to universal suicide prevention strategies (Table 1). These addressed 2 main program categories: (1) health promotion and suicide prevention through the use of educational websites and (2) health promotion and suicide prevention through awareness campaigns and social media psychoeducation. These programs were accessible to all participants. The types of websites identified were information-based, interactive, forums, and chats. Social media platforms included Facebook, Twitter, and personal and professional blogs. In general, the effects of these programs on perceptions and knowledge have been poorly evaluated. Universal suicide prevention strategies included the *SUPREME* (Suicide Prevention through Internet and Media-Based Mental Health Promotion) project [27], the *Storytelling* project [28], the *It Gets Better* project [29], the *Live Through This* project [30], and *Media-Based Prevention Messages* [31]. Although these different universal strategies could have some effects on the negative emotions of participants [32], they primarily play a potential role in reducing the stigma associated with suicide [30,33] and suicidal ideation and behavior [27,30,32] and increasing web-based suicide prevention knowledge [31,34,35], mental health literacy [32,33], and web-based help seeking [33,34]. Table 1 presents a description of the studies that referred to universal suicide prevention strategies.

**Table 1:** Universal suicide prevention strategies.

Type of program	Identified studies	Objectives	Programs, n	ICT^a^ used	Targeted population	Examples	Results and comments
Health promotion and suicide prevention through the use of educational websites	[27,28,30,32]	Improve mental health and well-being Educate and increase awareness of young people of mental health and suicide prevention Prevent stigma Encourage help seeking and service use by young people Promote protective factors	2	Websites Interactive modules Forums	Young people Young adults At-risk groups	“SUPREME^b^” project [27] and “Live Through This” [30]	Results in terms of reducing suicidal ideation and behavior, increasing mental health literacy, and increasing web-based help seeking seemed promising.
Health promotion and suicide prevention through awareness campaigns and social media psychoeducation	[29,31,33-36]	Increase social media users’ awareness of the existence of and issues associated with suicide and its prevention	4	Presenting, broadcasting and sharing messages on social media	Young people At-risk groups	“It Gets Better” project [29] and “Media-Based Prevention Messages” [31]	Results in terms of reducing suicidal ideation and behavior and improving knowledge, attitudes, and asking for help on the web seemed promising. However, further research was needed.

^a^ICT: information and communication technology.

^b^SUPREME: Suicide Prevention through Internet and Media-Based Mental Health Promotion.

### Selective Suicide Prevention Strategies

We identified 53 studies that dealt with selective suicide prevention strategies (Table 2). From these, 9 different selective suicide prevention program categories emerged, and each had multiple different outcomes. These strategies ranged from identifying people at risk to web-based self-management and training programs. Some were designed to be used alone, whereas others were part of a larger intervention program involving direct contact between suicidal individuals and suicide prevention resources. The programs targeted distressed individuals and groups at risk, that is, health professionals. ICT use varied according to the desired outcome. For example, algorithms were used to identify individuals at risk for suicide on the web, as advertised by Google AdWords, which is now known as Google Ads [37,38]; interactive websites were used to support the assessment and management of mental health disorders in collaboration with a mental health professional [39]; and training modules, web-based exercises, and multimedia presentations were used to enhance knowledge, understanding, and attitudes regarding suicide prevention and intervention [40-42]. Programs using algorithms to identify at-risk individuals seemed most promising for identifying people at risk for suicide versus those not at risk [37,38,43-58]. These algorithms analyzed suicide risk on the basis of different elements, including speech and linguistic characteristics, medical notes, search engine ad clicks, and profiles from web-based chat sessions or social media [37,38,43-58]. Although these algorithms helped identify individuals at risk for suicide, a clinical application of these algorithms is yet to be developed, as little is known about the effectiveness of these programs in increasing actual web-based and in-person help-seeking behaviors.

**Table 2:** Selective suicide prevention strategies.

Type of program	Identified studies	Objectives	Programs, n	ICT^a^ used	Targeted population	Examples	Results and comments
Identifying at-risk individuals through automatic analysis of their linguistic characteristics during face-to-face consultations	[44-47]	Identify individuals at risk for suicide and qualify risk level based on speech characteristics	2	Analysis of characteristics from observation of spoken language	Individuals presenting risk factors at assessment interviews	Analysis of speech and sound characteristics program [45-47]	This technology seems effective in distinguishing suicidal from nonsuicidal individuals. A clinical use of this technology is yet to be developed.
Identifying at-risk individuals through automatic analysis of medical file notes and/or research data	[54-57]	Identify individuals at risk for suicide and qualify risk level based on data (medical file or research data) from clinical observations by clinicians or researchers	1	Analysis of written language	Individuals presenting risk factors during assessment interviews	The “Safety-Net” program [56]	This technology seems effective in distinguishing suicidal from nonsuicidal individuals. A clinical use of this technology is yet to be developed.
Identifying at-risk individuals through automatic analysis of their linguistic characteristics during chat sessions or on written forms	[43,58]	Identify individuals at high risk for suicide based on characteristics of their words or responses	0	Analysis of written language	Individuals presenting risk factors during written exchanges	Suicide meaning making applied in specific contexts [58]	This technology seems promising. A clinical use of this technology is yet to be developed.
Identifying at-risk individuals through automatic analysis of their linguistic characteristics on social media	[48-53,59-62]	Examine writings of social media users to automatically identify individuals at risk for suicide and offer them proactive help	4	Analysis of written language	Social media users	Program to screen suicidal individuals based on tweets [52,59]	This technology is innovative. Further research is needed.
Identifying at-risk individuals through targeted ads on search engines	[37,38]	Identify suicidal individuals through their queries on search engines Suggest help resources	2	Search engine algorithm and targeted ads	General public	Web-based sentinel program [37]	The number of clicks on targeted ads serves as an indicator. There are too few results to date to evaluate these programs’ effectiveness in increasing the web-based help-seeking behaviors of suicidal individuals.
Web-based identification programs tailored to different groups	[63-69]	Identify vulnerable groups using mailing lists or websites to direct them toward help resources	3	Web-based standardized risk assessment questionnaires with response algorithms adapted to assessment results	Individuals at risk for suicide	EMPATHY^b^ [63] and HEAR^c^ for nurses [69]	This type of program allows identifying individuals at risk who have no contact with health services. Utilization rates for proposed resources seem encouraging.
Web-based mental health self-evaluation programs	[39,70]	Support evaluation and management of mental health problems in conjunction with a mental health professional	1	Interactive website Web-based evaluation tool Algorithm tool for redirecting to resources Development of a web-based personalized intervention plan	Individuals receiving support from a health professional	myGRaCE decision support system [39] and YouthCHAT [70]	Improves patient engagement in understanding their situation and in managing their treatment.
Web-based mental health self-management program with measures of impact on suicide risk	[71-81]	Facilitate web-based mood self-management and improve quality of life through a cognitive behavioral intervention	5	Interactive website Psychoeducation module Multimedia presentation Web-based exercises Discussion and consultation forum	Individuals with low-intensity mental health problems	MindSpot Clinic [77]	Program membership rates are relatively low. Individuals tend more often to experience a decrease in suicidal ideation and depression symptoms. Programs are offered alone or in conjunction with clinical follow-up by a professional. Results are hard to compare.
Web-based suicide prevention training programs	[40-42,82-87]	Improve knowledge, attitudes and suicide prevention practices of professionals and sentinels	5	Website Training modules Multimedia demonstration Web-based exercises	Professionals Sentinels	Question, Persuade, Refer, and Treat program [40,84]	These programs allow improving knowledge and attitudes. They seem less effective in changing intervention practices if not offered along with certain forms of face-to-face practices.

^a^ICT: information and communication technology.

^b^EMPATHY: Empowering a Multimodal Pathway Toward Healthy Youth.

^c^HEAR: Healer Education Assessment and Referral.

Using ICT to identify individuals at risk for suicide in school (from elementary school to medical school) or community settings has been associated with increased mental health literacy, help-seeking behavior, and use of help resources [63-65]. For example, in the Health Education Assessment and Referral (HEAR) program, medical students anonymously responded to a web-based questionnaire on suicide risk and various mental health issues. The program allowed identifying students at risk for suicide and referring them to a medical school psychiatrist or psychologist through a web-based platform. As a result, the use of medical schools’ mental health services increased from 11.5% to 15% over 4 years [64].

Our review also identified web-based self-assessment and self-management programs. For example, myGRaCE is a decision-making support system that combines service user self-assessment and practitioner expertise by comparing the user’s self-assessment against a practitioner’s assessment [39]. Most of the participants in this study agreed that myGRaCE helped them assess their personal security, understand what puts them in danger, and what areas they should change in their lives. Self-assessment programs are also a way to engage young people and adults by helping them understand their situation and identify ways to take control of their health [39,70]. As for web-based self-management programs, results showed that many programs could, in some cases, significantly reduce suicidal ideation [71-74] and increase chances of resorting to mental health interventions [75,76]. However, the rate of adherence to these types of programs remains low. Examples of these include MoodGYM [71], MindSpot Clinic [77], different types of internet-based cognitive behavioral therapy (iCBT), Thrive [73], the Sadness program [78], and CATCH-IT (Competent Adulthood Transition with Cognitive, Behavioral, Humanistic and Interpersonal Training) [74]. These programs were offered either alone or in combination with clinical follow-up. Users seemed to appreciate the mobile apps used for self-management and suicide prevention training. Many web-based suicide prevention programs have been tested for their efficacy. These programs were intended for gatekeepers who work with adolescents [82], gatekeepers in school settings [83], mental health professionals [40], health professionals in general [41,42,84], Veterans Affairs providers [85], and graduate students [86]. In some cases, face-to-face training developed more knowledge and suicide prevention skills than web-based training [84]. In addition, an increase in suicide prevention knowledge was often observed within the first months post training but often decreased over time [82]. Details of the studies that referred to selective suicide prevention strategies are presented in Table 2.

### Indicated Suicide Prevention Strategies

Regarding the indicated suicide prevention strategies, we selected 52 studies for our review and identified 9 program categories. These programs, presented in Table 3, were offered by health professionals or psychosocial help services and covered suicide risk assessment triage and monitoring, crisis intervention, low-intensity psychological interventions, psychotherapy for individuals at risk for suicide, and technological tools to support face-to-face interventions. The ICTs used for these programs included classification algorithms, chats and text messages, mobile apps, and interactive clinical intervention websites. Examples of these programs include the Mental Health eClinic [55], a web-based assessment and self-management program; Reframe-IT [88], an internet-based CBT program for high school students at risk for suicide; MYPLAN [89], a mobile phone safety plan app for supporting people at risk for suicide; dialectical behavioral therapy Coach Mobile [90], a CBT support intervention mobile phone app; and RAFT (Reconnecting After a Suicide Attempt) [91], a brief web-based outreach intervention for people who attempted suicide and who lost contact with services. In general, ICT-based self-assessment tools have been reported to be as effective in person as in hardcopy format in identifying suicide risk warning signs [92].

**Table 3:** Indicated suicide prevention strategies.

Type of program	Identified studies	Objectives	Programs, n	ICT^a^ used	Targeted population	Examples	Results and comments
Development of web-based suicide risk assessment tools	[92-97]	Propose suicide risk assessment tools administered electronically in clinical contexts	4	Electronic clinical instrument Response selection algorithm based on assessment results	Individuals at risk for suicide in contact with health services	Web-based Columbia Suicide Severity Rating Scale [93]	Electronic assessment tools seem as effective as hardcopy versions. They allow internet users at times to reveal their suicidal behaviors more easily
Use of artificial intelligence and machine learning to optimize completion time for suicide risk assessment tools	[98]	Reduce completion time for suicide risk assessment tools	1	CAT^b^	Individuals at risk for suicide in contact with health services	CAT [98]	This type of algorithm allows reducing the number of items needed to assess suicide risk.
Use of triage systems by ICT to assess suicide risk	[99]	Improve triage of individuals at risk for suicide who use health services	1	On-lining of clinical suicide risk assessment tools Risk-level classification algorithm based on scores Targeted offer of resources Automatic alert to the clinical team	Individuals at risk for suicide in contact with health services	Mental health eClinic [99]	This type of program allows identifying young people at risk for suicide and offering them clinical treatment more rapidly.
Crisis intervention via text messaging or internet chatting	[68,69,84,85]	Intervene in a crisis situation via text messaging or internet chatting	3	Text messaging Internet chatting Computerized system for processing text messages and chat exchanges	Young people are the priority target group of these interventions. They can, however, be used to reach other age groups	Kids Help Phone LIVECHAT Project [100] and RAFT^c^ [91]	Text messages and internet chat interventions seem a viable alternative to telephone interventions for certain groups. However, intervention strategies adapted to these modes of communication need to be developed.
Use of web-based publications by patients as intervention support material	[101,102]	Assess suicide risk and intervene using a person’s web-based discourse	1	Content of posts written on social networks	Suicidal individuals receiving mental health services	Patient’s social networking sites as a clinical tool [101]	The use of content written on social networks by patients allows a better understanding of the situation and helps perform a suicide risk assessment, especially in the case of patients who deny such behaviors.
Web-based management and low-intensity intervention for individuals at risk for suicide	[1,31,63,68,88,102-114]	Intervene with individuals at risk for suicide using a cognitive behavioral approach	7	Interactive website Web-based exercise module Discussion forum Multimedia presentation	General population Individuals referred to programs by clinicians	Reframe-IT [88], Fitmindkit [102], EMPATHY^d^ [63], and SMART^e^ Mental Health Project [110,113]	These programs bring about a small decrease in suicide risk among participants.
Mobile apps used as part of treatment follow-up with suicidal individuals	[90,115-121]	Support face-to-face clinical intervention Support aboriginal young people by complementing face-to-face intervention	2	Interactive mobile app Algorithm for providing responses and offering resources based on information provided by the user	Individuals receiving mental health services Aboriginal young people	DBT^f^ Coach Mobile [90] and AIMhi Stay Strong iPad [101]	These apps seem to help reduce the danger of suicide and the urgency of self-harm. Perceptions are positive, and apps allow reaching young people in isolated communities.
Use of web-based monitoring tools to improve psychological follow-up of individuals at risk for suicide	[107,122,123]	Conduct regular evaluations of individuals and their symptoms to inform and adjust intervention	3	Digitalization of screening tools Mobile app Contact with intervention team via automated alerts Automated sending of text messages	Individuals diagnosed with a mood disorder and receiving mental health services	Depression Project [123]	The use of an app facilitates disclosure of suicidal and self-harm behaviors. Regular monitoring of symptoms helps adjust intervention strategies.
Follow-up program by automatic text messages for individuals at risk for suicide	[91,124-126]	Offer tailored follow-up following a suicide attempt to increase treatment use and reduce suicidal behaviors and self-harm	3	Automated sending of predrafted text messages, including encouragement and appointment date reminders	Suicidal individuals receiving mental health services	Postattempt follow-up program by text messaging [124]	Users appreciate the text messages and deem them a good way of keeping in touch with care services. Help-seeking behaviors increase, and self-harm behaviors decrease.

^a^ICT: information and communication technology.

^b^CAT: computerized adaptive testing.

^c^RAFT: Reconnecting After a Suicide Attempt.

^d^EMPATHY: Empowering a Multimodal Pathway Toward Healthy Youth.

^e^SMART: Systematic Medical Appraisal, Referral and Treatment.

^f^DBT: dialectical behavioral therapy.

In some cases, web-based self-assessment tools, as opposed to face-to-face tools, seemed to facilitate the disclosure of suicidal behavior [93]. AI has also been shown to be effective in optimizing the web-based assessment of suicide behavior by using an item response-based computer-adaptive simulation to reduce the length of a suicide risk assessment tool [98]. As for ICT-based triage systems, the use of the Synergy Online System allowed young people to complete a web-based clinical assessment before a face-to-face or web-based clinical appointment, who were to be prioritized and contacted immediately in case of high suicide risk [99]. Web-based crisis interventions by chat or SMS text messaging have been on the rise and seem to be an alternative to phone interventions for some groups. Studies comparing chat services with SMS text messaging have shown similar results [127]. However, users of chat services, such as 113Online [128], were more likely to be at a high level of suicide crisis, had more mental health problems, were younger, and were more often to be women compared with crisis hotline users. In addition, interventions delivered through chat services were longer and more complicated, and fewer changes were observed in the individual’s emotional state [127,128]. Interventions have yet to be developed and adapted to this type of technology; the fact that interventions designed to be delivered by telephone were delivered by chat or SMS text messaging without being adapted to these other media was considered a major limitation [128]. Some studies also analyzed content posts by suicidal patients on social networking sites to better assess suicide risk, especially in patients denying suicidal behavior [101,129]. Although social media content helped with better understanding a person’s situation, there were many ethical concerns regarding this data collection method [129].

Self-management and iCBT programs seemed to reduce suicidal ideation. Unlike iCBT and self-management interventions addressing general mental health in the selective suicide prevention section, these interventions specifically addressed user suicide behaviors. Examples of these programs included Empowering a Multimodal Pathway Toward Healthy Youth [63], Reframe-IT [88], Fitmindkit [102], Safe conversation [103], Latitudes [103] and PrevenDep [104]. Intervention goals included developing basic problem-solving skills [88] and cognitive restructuring [105] to reduce suicide risk. Other ICT suicide prevention interventions include using computer alerts or mobile apps to apply a safety plan. These ranged from a system alerting the clinician not to forget to use the safety plan with a patient [130] to a mobile app of a personalized safety plan that patients could have on their phones at all times [89,115]. Research has demonstrated a good level of acceptability [131] for these interventions, but further evaluation of their effects on suicidal behaviors is necessary. Mobile apps have also been used to support therapeutic follow-up for suicidal patients [90,115,116], including for specific groups such as Aboriginals and Torres Strait Island Australians [117]. These mobile apps seemed to help reduce suicidal danger and self-harm [90,118], reach young people in isolated communities [132], and increase adherence to face-to-face follow-ups [119].

## Discussion

### Limitations

The rapid expansion of ICT use in suicide prevention has preceded the development of theoretical models to orient its methods and content and the accumulation of sufficient empirical research to indicate what is most helpful and what is not helpful. In this context, common sense has been the guiding principle, resulting in a plethora of suicide prevention activities being implemented with limited research evaluations of their effectiveness slowly following. Many of the most widely used ICT suicide prevention services have never been evaluated. Therefore, the published studies concern a small nonrandom sample of select interventions that a few researchers have been interested in studying. When little or no research has been published on ICT programs, this does not mean that they are not effective. Similarly, when there are promising empirical data on the benefits of a program, this does not mean that it is better or more useful than programs that have not been studied. Furthermore, we do not know if the *promising* results reported will stand the test of time, as more research is conducted using more rigorous research methodologies. Therefore, any conclusions drawn from the limited scientific publications must be considered preliminary and hopefully will be subject to verification in the future.

### Contributions

This scoping review on ICT use in suicide prevention shows that a large number of studies have been published in the past few years [1,3,5]. Our findings shed light on the use of ICT in different types of universal, selective, and indicated suicide prevention strategies.

Amid publications with the potential to reach a large population, there are only a few publications on the use of ICT in universal suicide prevention [27-35,133]. Our findings reveal that there are around 85% fewer studies on universal prevention strategies than those on selective and indicated strategies. The 10 publications we identified describe health promotion and suicide prevention through educational websites, web-based awareness campaigns, and social media [27-35,133]. The limited empirical findings suggest that these programs may play a role in increasing general mental health literacy and the incidence of help-seeking behavior, which are associated with reduced suicidal ideation and behavior. However, the effects of these programs on perceptions and knowledge have rarely been investigated. It is important to identify which characteristics of educational websites, awareness campaigns, and postings on social media are associated with positive changes in help seeking and reductions in suicidal ideations and behaviors. Furthermore, it is essential to develop effective means for identifying sites and postings that are helpful and notifying users about or orienting them toward internet content that may be of help to them.

Selective suicide prevention strategies using ICT consist of programs that identify specific subpopulations to offer specialized support to reduce suicide risk. They particularly targeted young people and various at-risk groups (eg, the lesbian, gay, bisexual, transgender, queer, and other community; sexual minorities; and aboriginal communities) through their profiles and linguistic characteristics (eg, chat sessions, web-based written forms, and social media) or by analyzing their medical records and research data. They also include targeted web-based advertising, web-based self-assessment and self-management, and web-based training. A larger published body of research indicates that these approaches are promising, particularly in controlled environments such as schools. School programs using ICT, such as HEAR, have been shown to have a positive impact on mental health help seeking and service use in youth [64]. Programs targeting specific subgroups of the general population have been shown to increase mental health literacy and the chances of using mental health services, but this effect seems to decrease over time [71,73,74,77,92]. Therefore, it is important to focus on the sustainability of the effects of programs that have an initial positive impact. The novelty of an intervention may be associated with greater effects. If novelty is a key feature of programs’ success, then either the programs need to be constantly changed and renewed to sustain their impact or the programs need to be continually replaced by new and different activities to ensure that people will continue to be helped over time. Therefore, both approaches may be proven to be unsustainable over time. This rapid effect can also give a false sense that resorting to web-based strategies is sufficient. However, web-based help does not replace face-to-face or direct support and care from trained professionals. These different selective suicide prevention strategies should only be used in addition to face-to-face type of help and interventions. Moreover, our empirical findings show that more research is required to better distinguish between false positives and false negatives in these web-based identification techniques. It is of utmost importance to help identify at-risk groups and avoid discarding individuals who are assessed as false negatives too quickly. A false impression of security can put these individuals at a greater risk.

Where indicated suicide prevention strategies are concerned, programs vary, including using ICT in suicide risk assessment triage, monitoring suicide risk, crisis intervention activities, and psychotherapy. The programs were offered on the web only (eg, websites, mobile phone apps, and chats), but they were sometimes supported by face-to-face interventions. In some instances, they were found to be efficient (eg, shortened web-based assessments), and sometimes they were time-consuming (eg, interventions were longer in chat sessions). ICT-based and self-management interventions seem promising, as they address suicidal behaviors directly and offer alternatives for coping with suicidal thoughts by developing problem-solving skills [88,90-92]. In addition, specific at-risk groups in isolated communities where other services are not available may benefit from mobile apps used for patient follow-up.

### Ethical Considerations to Address in the Future

Many of the studies included in this review raised security and ethical concerns regarding web-based suicide prevention practices. Ethical concerns range from a lack of training and web-based moderators’ skills to the lack of an evidence-based framework providing guidelines for the secure use of ICT [35]. As mentioned by Robinson et al [35], it is ethically necessary to provide security protocols and a clear code of ethics for safe web-based intervention. Another ethical concern is that all individuals have the right to privacy, including web privacy [101,129]. Thus, there is reason to question whether the content analysis of web-based social media posts, emails, and other web-based sources of information is an ethical research endeavor if informed consent is not obtained from the individuals who have posted the information [101,129]. Beyond security and privacy, there are many other ethical concerns regarding web-based surveillance, informed consent, communication, controls, and disclosure [134,135]. For example, a proper ethical assessment of risks and benefits to the use of different ICT strategies is rarely, if almost never, considered in the development process. There is also a major ethical concern regarding reducing or eliminating in-person services and replacing them with insufficient web-based solutions that may appear to present better cost-effectiveness without proper in-depth assessment. With this concern, web-based interventions should always be combined with in-person formal help and intervention services. This raises many concerns and provides plenty of grounds for further research aimed at ensuring safer and more ethical use of ICT in suicide prevention.

### Conclusions

As the number of studies on ICT use in suicide prevention is growing, the published literature needs to be reviewed regularly. This scoping review shows that ICT use in suicide prevention provides an interactive, personalized, readily available, and accessible approach to reach various populations for identification of at-risk individuals and to provide support. ICT may provide a sense of *being connected* to people who are otherwise isolated and reluctant to use offline services. Promising published findings on web-based intervention content includes psychoeducation and skills training. As digital help proliferates, one should consider whether this means that help-seeking and suicide prevention activities will replace traditional offline services. However, in some areas where radical changes were expected, existing modalities continue to be used (eg, individuals did not stop using in-person services when telephone phone crisis lines appeared, and these telephone lines reached a different audience and complemented existing services). The extent to which ICT will become the main source of suicide prevention activities will depend upon its efficacy in helping people, compared with and as a complement to existing services and activities.

Although there is a growing body of evidence regarding ICT use in suicide prevention, program evaluation is still lacking. There is a need for more research evaluating and comparing the impacts of various ICT strategies in different contexts, understanding the profiles of individuals at risk of suicide who use ICT, and web-based help-seeking behaviors. We also need to better understand the impact of ICT on individuals who are bereaved by suicide. Moreover, ethics and security concerns regarding web-based suicide prevention have been the focus of very limited research and need to be addressed in future studies.

Furthermore, service users, providers, and managers from private or public systems should be informed as of now and updated regularly on the benefits and risks of ICT use in health and social services. This information should include (1) effectiveness in various well-described contexts and potential unexpected iatrogenic effects, (2) cost-benefit relationship to the best comparator, (3) access, (4) acceptability and ethical concerns, (5) security, and (6) implementation. Concerning implementation, quality standards should be similar to those of Improving Access to Psychological Therapies standards set by the United Kingdom for access to psychotherapy. These standards are (1) a model of care, (2) access, (3) evidence-based interventions, (4) outcome-based measurement, and (5) the provider’s training and supervision. International health and social technology assessment agencies should therefore consider developing guidelines and a system of voluntary accreditation for ICT use in suicide prevention. National and regional public health and social services may require that, before commissioning an ICT for suicide prevention or mental health care, their Health Technology Agency recommends ICT based on its efficacy, efficiency, safety, acceptability, and feasibility in the context of jurisdiction.

## Appendix

Multimedia Appendix 1 Search strategy syntax specific to each consulted database.

## Abbreviations

AI: artificial intelligence
CBT: cognitive behavioral therapy
HEAR: Health Education Assessment and Referral
iCBT: internet-based cognitive behavioral therapy
ICT: information and communication technology
PICO: population, intervention, comparison, and outcomes
PRISMA: Preferred Reporting Items for Systematic Reviews and Meta-Analyses

## References

[1] Larsen ME, Nicholas J, Christensen H (2016). A systematic assessment of smartphone tools for suicide prevention. PLoS One.

[2] van Spijker BA, Werner-Seidler A, Batterham PJ, Mackinnon A, Calear AL, Gosling JA, Reynolds J, Kerkhof AJ, Solomon D, Shand F, Christensen H (2018). Effectiveness of a web-based self-help program for suicidal thinking in an Australian community sample: randomized controlled trial. J Med Internet Res.

[3] Perry Y, Werner-Seidler A, Calear A, Christensen H (2016). Web-based and mobile suicide prevention interventions for young people: a systematic review. J Can Acad.

[4] de la Torre I, Castillo G, Arambarri J, López-Coronado M, Franco MA (2017). Mobile apps for suicide prevention: review of virtual stores and literature. JMIR mHealth and uHealth.

[5] Robinson J, Cox G, Bailey E, Hetrick S, Rodrigues M, Fisher S, Herrman H (2016). Social media and suicide prevention: a systematic review. Early Interv Psychiatry.

[6] Robinson J, Bailey E, Witt K, Stefanac N, Milner A, Currier D, Pirkis J, Condron P, Hetrick S (2018). What works in youth suicide prevention? A systematic review and meta-analysis. EClinicalMedicine.

[7] van Spijker BAJ, van Straten A, Kerkhof AJ (2014). Effectiveness of online self-help for suicidal thoughts: results of a randomised controlled trial. PLoS ONE.

[8] Wagner B, Horn AB, Maercker A (2014). Internet-based versus face-to-face cognitive-behavioral intervention for depression: a randomized controlled non-inferiority trial. J Affect Disord.

[9] Organisation mondiale de la Santé (2014). Prévention du suicide: l'état d'urgence mondial.

[10] Le grand dictionnaire terminologique (GDT). Office québécois de la langue française.

[11] Arksey H, O'Malley L (2005). Scoping studies: towards a methodological framework. International Journal of Social Research Methodology.

[12] Levac D, Colquhoun H, O'Brien K (2010). Scoping studies: advancing the methodology. Implementation Science.

[13] Mishara B, Kerkhof AJFM (2013). Suicide Prevention and New Technologies: Evidence-Based Practice.

[14] Donker T, Petrie K, Proudfoot J, Clarke J, Birch M, Christensen H (2013). Smartphones for smarter delivery of mental health programs: a systematic review. J Med Internet Res.

[15] Grist R, Porter J, Stallard P (2017). Mental Health Mobile Apps for Preadolescents and Adolescents: A Systematic Review. J Med Internet Res.

[16] Shamseer L, Moher D, Clarke M, Ghersi D, Liberati A, Petticrew M, Shekelle P, Stewart LA, the PRISMA Group (2015). Preferred reporting items for systematic review and meta-analysis protocols (PRISMA-P) 2015: elaboration and explanation. BMJ.

[17] Methely A, Campbell S, Chew-Graham C, McNally R, Cheraghi-Sohi S (2014). PICO, PICOS and SPIDER: a comparison study of specificity and sensitivity in three search tools for qualitative systematic reviews. BMC Health Services Research.

[18] Straus SE, Tetroe J, Graham ID, Zwarenstein M, Bhattacharyya O, Shepperd S (2010). Monitoring use of knowledge and evaluating outcomes. Canadian Medical Association Journal.

[19] Pourmand A, Roberon J, Caggiula A, Monsalve N, Rahimi M, Torres-Llenza V (2019). Social media and suicide: a review of technology-based epidemiology and risk assessment. Telemedicine and e-Health.

[20] Bartels EM (2013). How to perform a systmatic search. Best Practice & Research Clinical Rheumatology.

[21] McHugh ML (2012). Interrater reliability: the kappa statistic. Biochem Med (Zagreb).

[22] Mishara B, Chagnon F, Daigle M, Balan B, Raymond S, Marcoux I, Bardon C, Campbell J, Berman A (2007). Comparing models of helper behavior to actual practice in telephone crisis intervention: A silent monitoring study of calls to the US 1-800-SUICIDE network. Suicide and Life-Threatening Behavior.

[23] Mishara B, Daigle M, Lester D (2001). Helplines crisis intervention services: challenges for the future. Suicide Prevention: resources for the millennium.

[24] Mishara BL, Daigle M, Bardon C, Chagnon F, Balan B, Raymond S, Campbell J (2016). Comparison of the Effects of Telephone Suicide Prevention Help by Volunteers and Professional Paid Staff: Results from Studies in the USA and Quebec, Canada. Suicide Life Threat Behav.

[25] Henschke N, Ostelo R, Vlaeven S, Morley S, Assendelft W, Main C (2010). Behavioural treatment for chronic low-back pain. Cochrane Database System Reviews.

[26] Schünemann H, Vist G, Higgins J, Santesso N, Deeks J, Glasziou P, Akl E, Guyatt G, Higgins JPT, Thomas J, Chandler J, Cumpston M, Li T, Page MJ, Welch VA, on behalf of the Cochrane GRADEing Methods Group (2019). Interpreting results and drawing conclusions. Cochrane Handbook for Systematic Reviews of Interventions, Second Edition.

[27] Carli V, Courtet P (2016). Preventing suicidality through online tools: The SUPREME Project. Understanding Suicide: from Diagnosis to Personalized Treatment.

[28] Wexler L, Gubrium A, Griffin M, DiFulvio G (2012). Promoting Positive Youth Development and Highlighting Reasons for Living in Northwest Alaska Through Digital Storytelling. Health Promotion Practice.

[29] Hurley, SJ (2014). Public pedagogy and the experience of video creators in the It Gets Better Project. Libraries digital conservancy.

[30] Rogers ML, Schneider ME, Gai AR, Gorday JY, Joiner TE (2018). Evaluation of two web-based interventions in reducing the stigma of suicide. Behaviour Research and Therapy.

[31] Robinson J, Bailey E, Hetrick S, Paix S, O'Donnell M, Cox G, Ftanou M, Skehan J (2017). Developing Social Media-Based Suicide Prevention Messages in Partnership With Young People: Exploratory Study. JMIR Ment Health.

[32] Till B, Tran US, Voracek M, Niederkrotenthaler T (2018). Beneficial and harmful effects of educative suicide prevention websites: randomised controlled trial exploring Papageno . Werther effects. Br J Psychiatry.

[33] Taylor-Rodgers E, Batterham PJ (2014). Evaluation of an online psychoeducation intervention to promote mental health help seeking attitudes and intentions among young adults: randomised controlled trial. J Affect Disord.

[34] Berger J (2013). Adolescents on the lookout for suicidal friends on social networking sites. Digital Repository at the University of Maryland.

[35] Robinson J, Rodrigues M, Fisher S, Bailey E, Herrman H (2015). Social media and suicide prevention: findings from a stakeholder survey. Shanghai Arch Psychiatry.

[36] Chen JI, Smolenski DJ, Dobscha SK, Bush NE, Denneson LM (2018). Correlates of Mental Health Smartphone Application Use among Patients with Suicidal Ideation. Journal of Technology in Human Services.

[37] Sueki H, Ito J (2015). Suicide prevention through online gatekeeping using search advertising techniques: a feasibility study. Crisis.

[38] Liu NH, Contreras O, Muñoz RF, Leykin Y (2014). Assessing Suicide Attempts and Depression Among Chinese Speakers Over the Internet. Crisis.

[39] Buckingham CD, Adams A, Vail L, Kumar A, Ahmed A, Whelan A, Karasouli E (2015). Integrating service user and practitioner expertise within a web-based system for collaborative mental-health risk and safety management. Patient Education and Counseling.

[40] Gryglewicz K, Chen JI, Romero GD, Karver MS, Witmeier M (2017). Online Suicide Risk Assessment and Management Training. Crisis.

[41] Ryan K, Tindall C, Strudwick G (2017). Enhancing Key Competencies of Health Professionals in the Assessment and Care of Adults at Risk of Suicide Through Education and Technology. Clinical Nurse Specialist.

[42] LeCloux M (2018). The development of a brief suicide screening and risk assessment training webinar for rural primary care practices. Journal of Rural Mental Health.

[43] Passos IC, Mwangi B, Cao B, Hamilton JE, Wu M, Zhang XY, Zunta-Soares GB, Quevedo J, Kauer-Sant'Anna M, Kapczinski F, Soares JC (2016). Identifying a clinical signature of suicidality among patients with mood disorders: A pilot study using a machine learning approach. J Affect Disord.

[44] Cook BL, Progovac AM, Chen P, Mullin B, Hou S, Baca-Garcia E (2016). Novel Use of Natural Language Processing (NLP) to Predict Suicidal Ideation and Psychiatric Symptoms in a Text-Based Mental Health Intervention in Madrid. Computational and Mathematical Methods in Medicine.

[45] Pestian J, Grupp-Phelan J, Bretonnel CK, Meyers G, Richey L, Matykiewicz P, Sorter M (2016). A controlled trial using natural language processing to examine the language of suicidal adolescents in the emergency department. Suicide & Life-Threatening Behavior.

[46] Pestian J, Sorter M, Connolly B, Cohen K, McCullumsmith C, Gee J, Morency L, Scherer S, Rohlfs L (2017). A machine learning approach to identifying the thought markers of suicidal subjects: a prospective multicenter trial. Suicide and Life-Threatening Behavior.

[47] Venek V, Scherer S, Morency L, Rizzo AS, Pestian J (2017). Adolescent Suicidal Risk Assessment in Clinician-Patient Interaction. IEEE Trans. Affective Comput.

[48] Braithwaite SR, Giraud-Carrier C, West J, Barnes MD, Hanson CL (2016). Validating Machine Learning Algorithms for Twitter Data Against Established Measures of Suicidality. JMIR Ment Health.

[49] Cheng Q, Li TM, Kwok C, Zhu T, Yip PS (2017). Assessing Suicide Risk and Emotional Distress in Chinese Social Media: A Text Mining and Machine Learning Study. J Med Internet Res.

[50] Guan L, Hao B, Cheng Q, Yip PS, Zhu T (2015). Identifying Chinese Microblog Users With High Suicide Probability Using Internet-Based Profile and Linguistic Features: Classification Model. JMIR Mental Health.

[51] Lv M, Li A, Liu T, Zhu T (2015). Creating a Chinese suicide dictionary for identifying suicide risk on social media. PeerJ.

[52] O'Dea B, Larsen ME, Batterham PJ, Calear AL, Christensen H (2017). A Linguistic Analysis of Suicide-Related Twitter Posts. Crisis.

[53] Ren F, Kang X, Quan C (2016). Examining Accumulated Emotional Traits in Suicide Blogs With an Emotion Topic Model. IEEE J. Biomed. Health Inform.

[54] Hettige NC, Nguyen TB, Yuan C, Rajakulendran T, Baddour J, Bhagwat N, Bani-Fatemi A, Voineskos AN, Mallar Chakravarty M, De Luca V (2017). Classification of suicide attempters in schizophrenia using sociocultural and clinical features: A machine learning approach. General Hospital Psychiatry.

[55] Batterham PJ, van Spijker BAJ, Mackinnon AJ, Calear AL, Wong Q, Christensen H (2018). Consistency of trajectories of suicidal ideation and depression symptoms: Evidence from a randomized controlled trial. Depress Anxiety.

[56] Roaten K, Johnson C, Genzel R, Khan F, North CS (2018). Development and Implementation of a Universal Suicide Risk Screening Program in a Safety-Net Hospital System. The Joint Commission Journal on Quality and Patient Safety.

[57] Adamou M, Antoniou G, Greasidou E, Lagani V, Charonyktakis P, Tsamardinos I, Doyle M (2019). Toward Automatic Risk Assessment to Support Suicide Prevention. Crisis.

[58] Silvén Hagström A (2016). ‘Suicide stigma’ renegotiated: Storytelling, social support and resistance in an Internet-based community for the young suicide-bereaved. Qualitative Social Work.

[59] Burnap P, Colombo G, Amery R, Hodorog A, Scourfield J (2017). Multi-class machine classification of suicide-related communication on Twitter. Online Soc Netw Media.

[60] Du J, Zhang Y, Luo J, Jia Y, Wei Q, Tao C, Xu H (2018). Extracting psychiatric stressors for suicide from social media using deep learning. BMC Med Inform Decis Mak(Suppl 2).

[61] Coppersmith G, Leary R, Crutchley P, Fine A (2018). Natural Language Processing of Social Media as Screening for Suicide Risk. Biomed Inform Insights.

[62] Jung H, Park H, Song T (2017). Ontology-Based Approach to Social Data Sentiment Analysis: Detection of Adolescent Depression Signals. J Med Internet Res.

[63] Silverstone PH, Bercov M, Suen VYM, Allen A, Cribben I, Goodrick J, Henry S, Pryce C, Langstraat P, Rittenbach K, Chakraborty S, Engles RC, McCabe C (2017). Long-term Results from the Empowering a Multimodal Pathway Toward Healthy Youth Program, a Multimodal School-Based Approach, Show Marked Reductions in Suicidality, Depression, and Anxiety in 6,227 Students in Grades 6–12 (Aged 11–18). Front. Psychiatry.

[64] Downs N, Feng W, Kirby B, McGuire T, Moutier C, Norcross W, Norman M, Young I, Zisook S (2014). Listening to Depression and Suicide Risk in Medical Students: the Healer Education Assessment and Referral (HEAR) Program. Acad Psychiatry.

[65] Haskins J, Carson JG, Chang CH, Kirshnit C, Link DP, Navarra L, Scher LM, Sciolla AF, Uppington J, Yellowlees P (2015). The Suicide Prevention, Depression Awareness, and Clinical Engagement Program for Faculty and Residents at the University of California, Davis Health System. Acad Psychiatry.

[66] Williams D (2016). Consensus-based strategies for using social networking to reduce suicide risk: a Delphi study. Doctoral Dissertation.

[67] King CA, Eisenberg D, Zheng K, Czyz E, Kramer A, Horwitz A, Chermack S (2015). Online suicide risk screening and intervention with college students: a pilot randomized controlled trial. J Consult Clin Psychol.

[68] Silverstone PH, Bercov M, Suen VYM, Allen A, Cribben I, Goodrick J, Henry S, Pryce C, Langstraat P, Rittenbach K, Chakraborty S, Engels RC, McCabe C (2015). Initial Findings from a Novel School-Based Program, EMPATHY, Which May Help Reduce Depression and Suicidality in Youth. PLoS ONE.

[69] Davidson JE, Zisook S, Kirby B, DeMichele G, Norcross W (2018). Suicide Prevention: a healer education and referral program for nurses. JONA: The Journal of Nursing Administration.

[70] Goodyear-Smith F, Martel R, Darragh M, Warren J, Thabrew H, Clark TC (2017). Screening for risky behaviour and mental health in young people: the YouthCHAT programme. Public Health Rev.

[71] Christensen H, Farrer L, Batterham PJ, Mackinnon A, Griffiths KM, Donker T (2013). The effect of a web-based depression intervention on suicide ideation: secondary outcome from a randomised controlled trial in a helpline. BMJ Open.

[72] Mewton L, Andrews G (2015). Cognitive behaviour therapy via the internet for depression: a useful strategy to reduce suicidal ideation. J Affect Disord.

[73] Whiteside U, Richards J, Steinfeld B, Simon G, Caka S, Tachibana C, Stuckey S, Ludman E (2014). Online cognitive behavioral therapy for depressed primary care patients: a pilot feasibility project. Perm J.

[74] Saulsberry A, Marko-Holguin M, Blomeke K, Hinkle C, Fogel J, Gladstone T, Bell C, Reinecke M, Corden M, Van Voorhees BW (2013). Randomized Clinical Trial of a Primary Care Internet-based Intervention to Prevent Adolescent Depression: One-year Outcomes. J Can Acad Child Adolesc Psychiatry.

[75] Guille C, Zhao Z, Krystal J, Nichols B, Brady K, Sen S (2015). Web-Based Cognitive Behavioral Therapy Intervention for the Prevention of Suicidal Ideation in Medical Interns: A Randomized Clinical Trial. JAMA Psychiatry.

[76] Hedman E, Ljótsson B, Kaldo V, Hesser H, El Alaoui S, Kraepelien M, Andersson E, Rück C, Svanborg C, Andersson G, Lindefors N (2014). Effectiveness of Internet-based cognitive behaviour therapy for depression in routine psychiatric care. Journal of Affective Disorders.

[77] Nielssen O, Dear BF, Staples LG, Dear R, Ryan K, Purtell C, Titov N (2015). Procedures for risk management and a review of crisis referrals from the MindSpot Clinic, a national service for the remote assessment and treatment of anxiety and depression. BMC Psychiatry.

[78] Williams AD, Andrews G (2013). The Effectiveness of Internet Cognitive Behavioural Therapy (iCBT) for Depression in Primary Care: A Quality Assurance Study. PLoS ONE.

[79] Lara MA, Tiburcio M, Aguilar AA, Sánchez-Solís A (2014). A four-year experience with a Web-based self-help intervention for depressive symptoms in Mexico. Rev Panam Salud Publica.

[80] Gleeson J, Lederman R, Wadley G, Bendall S, McGorry P, Alvarez-Jimenez M (2014). Safety and privacy outcomes from a moderated online social therapy for young people with first-episode psychosis. Psychiatric services.

[81] Whiteside U, Lungu A, Richards J, Simon GE, Clingan S, Siler J, Snyder L, Ludman E (2014). Designing Messaging to Engage Patients in an Online Suicide Prevention Intervention: Survey Results From Patients With Current Suicidal Ideation. J Med Internet Res.

[82] Ghoncheh R, Gould MS, Twisk JW, Kerkhof AJ, Koot HM (2016). Efficacy of Adolescent Suicide Prevention E-Learning Modules for Gatekeepers: A Randomized Controlled Trial. JMIR Ment Health.

[83] Lamis DA, Underwood M, D'Amore N (2017). Outcomes of a Suicide Prevention Gatekeeper Training Program Among School Personnel. Crisis.

[84] Smith AR, Silva C, Covington DW, Joiner TE (2014). An assessment of suicide-related knowledge and skills among health professionals. Health Psychology.

[85] Marshall E, York J, Magruder K, Yeager D, Knapp R, De Santis ML, Burriss L, Mauldin M, Sulkowski S, Pope C, Jobes DA (2014). Implementation of Online Suicide-Specific Training for VA Providers. Acad Psychiatry.

[86] Krieg C (2017). Can demonstration enhance the effects of an online risk assessment training workshop? Doctoral dissertation, Counseling Psychology. Arizona State University. ASU Library. Digital Repository.

[87] Rigsbee N (2015). Suicide assessment training in counselor education. Doctoral dissertation. University of New Mexico. UNM Digital Repository.

[88] Robinson J, Hetrick S, Cox G, Bendall S, Yuen HP, Yung A, Pirkis J (2016). Can an Internet-based intervention reduce suicidal ideation, depression and hopelessness among secondary school students: results from a pilot study. Early Interv Psychiatry.

[89] Skovgaard Larsen JL, Frandsen H, Erlangsen A (2016). MYPLAN – A Mobile Phone Application for Supporting People at Risk of Suicide. Crisis.

[90] Rizvi S, Hughes C, Thomas M (2016). The DBT Coach mobile application as an adjunct to treatment for suicidal and self-injuring individuals with borderline personality disorder: a preliminary evaluation and challenges to client utilization. Psychological Services.

[91] Larsen ME, Shand F, Morley K, Batterham PJ, Petrie K, Reda B, Berrouiguet S, Haber PS, Carter G, Christensen H (2017). A Mobile Text Message Intervention to Reduce Repeat Suicidal Episodes: Design and Development of Reconnecting After a Suicide Attempt (RAFT). JMIR Ment Health.

[92] AL-Asadi AM, Klein B, Meyer D (2015). Multiple Comorbidities of 21 Psychological Disorders and Relationships With Psychosocial Variables: A Study of the Online Assessment and Diagnostic System Within a Web-Based Population. J Med Internet Res.

[93] Hesdorffer DC, French JA, Posner K, DiVentura B, Pollard JR, Sperling MR, Harden CL, Krauss GL, Kanner AM (2013). Suicidal ideation and behavior screening in intractable focal epilepsy eligible for drug trials. Epilepsia.

[94] Best P, Foye U, Taylor B, Hazlett D, Manktelow R (2013). Online interactive suicide support services: quality and accessibility. Mental Health Review Journal.

[95] Flamarique I, Santosh P, Zuddas A, Arango C, Purper-Ouakil D, Hoekstra P, Coghill D, Schulze U, Dittmann R, Buitelaar J, Lievesley K, Frongia R, Llorente C, Méndez I, Sala R, Fiori F, Castro-Fornieles J, STOP consortium (2016). Development and psychometric properties of the Suicidality: Treatment Occurring in Paediatrics (STOP) Suicidality Assessment Scale (STOP-SAS) in children and adolescents. BMC pediatrics.

[96] Hirschhorn E (2017). Integrating technology into collaborative suicide risk assessment: comparing electronic and paper-and-pencil versions of the suicide status form. Doctoral dissertation.

[97] AL-Asadi AM, Klein B, Meyer D (2014). Comorbidity Structure of Psychological Disorders in the Online e-PASS Data as Predictors of Psychosocial Adjustment Measures: Psychological Distress, Adequate Social Support, Self-Confidence, Quality of Life, and Suicidal Ideation. J Med Internet Res.

[98] De Beurs DP, de Vries AL, de Groot MH, de Keijser J, Kerkhof AJ (2014). Applying Computer Adaptive Testing to Optimize Online Assessment of Suicidal Behavior: A Simulation Study. J Med Internet Res.

[99] Iorfino F, Davenport TA, Ospina-Pinillos L, Hermens DF, Cross S, Burns J, Hickie IB (2017). Using New and Emerging Technologies to Identify and Respond to Suicidality Among Help-Seeking Young People: A Cross-Sectional Study. J Med Internet Res.

[100] Haner D, Pepler D (2016). "Live Chat" Clients at Kids Help Phone: Individual Characteristics and Problem Topics. J Can Acad Child Adolesc Psychiatry.

[101] Bennett A, Pourmand A, Shokoohi H, Shesser R, Sanchez J, Joyce J (2014). Impacts of Social Networking Sites on Patient Care in the Emergency Department. Telemedicine and e-Health.

[102] Batterham PJ, Calear AL, Farrer L, McCallum SM, Cheng VWS (2018). FitMindKit : Randomised controlled trial of an automatically tailored online program for mood, anxiety, substance use and suicidality. Internet Interventions.

[103] Rice S, Robinson J, Bendall S, Hetrick S, Cox G, Bailey E, Gleeson J, Alvarez-Jimenez M (2016). Online and Social Media Suicide Prevention Interventions for Young People: A Focus on Implementation and Moderation. J Can Acad Child Adolesc Psychiatry.

[104] Bresó A, Martínez-Miranda J, Botella C, Baños R, García-Gómez J (2016). Usability and acceptability assessment of an empathic virtual agent to prevent major depression. Expert Systems: International Journal of Knowledge Engineering and Neural Networks.

[105] Madsen T, van Spijker B, Karstoft K, Nordentoft M, Kerkhof AJ (2016). Trajectories of Suicidal Ideation in People Seeking Web-Based Help for Suicidality: Secondary Analysis of a Dutch Randomized Controlled Trial. J Med Internet Res.

[106] Hetrick SE, Goodall J, Yuen HP, Davey CG, Parker AG, Robinson J, Rickwood DJ, McRoberts A, Sanci L, Gunn J, Rice S, Simmons MB (2017). Comprehensive Online Self-Monitoring to Support Clinicians Manage Risk of Suicide in Youth Depression. Crisis.

[107] Hetrick SE, Yuen HP, Bailey E, Cox GR, Templer K, Rice SM, Bendall S, Robinson J (2017). Internet-based cognitive behavioural therapy for young people with suicide-related behaviour (Reframe-IT): a randomised controlled trial. Evid Based Mental Health.

[108] Batterham PJ, Christensen H, Mackinnon AJ, Gosling JA, Thorndike FP, Ritterband LM, Glozier N, Griffiths KM (2018). Trajectories of change and long-term outcomes in a randomised controlled trial of internet-based insomnia treatment to prevent depression. BJPsych open.

[109] Wilksch SM, O'Shea A, Wade TD (2018). Depressive symptoms, alcohol and other drug use, and suicide risk: Prevention and treatment effects from a two-country online eating disorder risk reduction trial. Int J Eat Disord.

[110] Maulik PK, Kallakuri S, Devarapalli S (2018). Operational challenges in conducting a community-based technology-enabled mental health services delivery model for rural India: Experiences from the SMART Mental Health Project. Wellcome Open Res.

[111] Reins JA, Boß L, Lehr D, Berking M, Ebert DD (2019). The more I got, the less I need? Efficacy of Internet-based guided self-help compared to online psychoeducation for major depressive disorder. Journal of Affective Disorders.

[112] Richards D, Duffy D, Burke J, Anderson M, Connell S, Timulak L (2018). Supported Internet-Delivered Cognitive Behavior Treatment for Adults with Severe Depressive Symptoms: A Secondary Analysis. JMIR Ment Health.

[113] Tewari A, Kallakuri S, Devarapalli S, Jha V, Patel A, Maulik PK (2017). Process evaluation of the systematic medical appraisal, referral and treatment (SMART) mental health project in rural India. BMC Psychiatry.

[114] Wilks CR, Lungu A, Ang SY, Matsumiya B, Yin Q, Linehan MM (2018). A randomized controlled trial of an Internet delivered dialectical behavior therapy skills training for suicidal and heavy episodic drinkers. Journal of Affective Disorders.

[115] Labelle R, Bibaud-De SA, Leblanc F, Mishara B, Kerkhof AJFM (2013). Innovating to treat depressionprevent suicide: the IPhone@ PSY ASSISTANCE application. Suicide Prevention and New Technologies: Evidence-Based Practices.

[116] Bush NE, Dobscha SK, Crumpton R, Denneson LM, Hoffman JE, Crain A, Cromer R, Kinn JT (2015). A Virtual Hope Box smartphone app as an accessory to therapy: proof-of-concept in a clinical sample of veterans. Suicide Life Threat Behav.

[117] Povey J, Mills PPJR, Dingwall KM, Lowell A, Singer J, Rotumah D, Bennett-Levy J, Nagel T (2016). Acceptability of Mental Health Apps for Aboriginal and Torres Strait Islander Australians: A Qualitative Study. J Med Internet Res.

[118] Mackie C, Dunn N, MacLean S, Testa V, Heisel M, Hatcher S (2017). A qualitative study of a blended therapy using problem solving therapy with a customised smartphone app in men who present to hospital with intentional self-harm. Evid Based Mental Health.

[119] Mok K, Jorm AF, Pirkis J (2016). Who Goes Online for Suicide-Related Reasons?. Crisis.

[120] Hetrick SE, Robinson J, Burge E, Blandon R, Mobilio B, Rice SM, Simmons MB, Alvarez-Jimenez M, Goodrich S, Davey CG (2018). Youth Codesign of a Mobile Phone App to Facilitate Self-Monitoring and Management of Mood Symptoms in Young People With Major Depression, Suicidal Ideation, and Self-Harm. JMIR Ment Health.

[121] O'Toole M, Arendt M, Pedersen C (2019). Testing an app-assisted treatment for suicide prevention in a randomized controlled trial: effects on suicide risk and depression. Behavior Therapy.

[122] Hetrick SE, Dellosa MK, Simmons MB, Phillips L (2014). Development and pilot testing of an online monitoring tool of depression symptoms and side effects for young people being treated for depression. Early Intervention in Psychiatry.

[123] Chen RY, Feltes JR, Tzeng WS, Lu ZY, Pan M, Zhao N, Talkin R, Javaherian K, Glowinski A, Ross W (2017). Phone-Based Interventions in Adolescent Psychiatry: A Perspective and Proof of Concept Pilot Study With a Focus on Depression and Autism. JMIR Res Protoc.

[124] Berrouiguet S, Gravey M, Le Galudec M, Alavi Z, Walter M (2014). Post-acute crisis text messaging outreach for suicide prevention: A pilot study. Psychiatry Research.

[125] Kodama T, Syouji H, Takaki S, Fujimoto H, Ishikawa S, Fukutake M, Taira M, Hashimoto T (2016). Text messaging for psychiatric outpatients: effect on help-seeking and self-harming behaviors. Journal of Psychosocial Nursing and Mental Health Services.

[126] Owens C, Charles N (2016). Implementation of a text-messaging intervention for adolescents who self-harm (TeenTEXT): a feasibility study using normalisation process theory. Child and Adolescent Psychiatry and Mental Health.

[127] Predmore Z, Ramchand R, Ayer L, Kotzias V, Engel C, Ebener P, Kemp JE, Karras E, Haas GL (2017). Expanding Suicide Crisis Services to Text and Chat. Crisis.

[128] Mokkenstorm JK, Eikelenboom M, Huisman A, Wiebenga J, Gilissen R, Kerkhof AJFM, Smit JH (2016). Evaluation of the 113Online Suicide Prevention Crisis Chat Service: Outcomes, Helper Behaviors and Comparison to Telephone Hotlines. Suicide Life Threat Behav.

[129] Ahuja AK, Biesaga K, Sudak DM, Draper J, Womble A (2014). Suicide on facebook. J Psychiatr Pract.

[130] Reyes-Portillo JA, Chin EM, Toso-Salman J, Blake Turner J, Vawdrey D, Mufson L (2018). Using Electronic Health Record Alerts to Increase Safety Planning with Youth At-Risk for Suicide: A Non-randomized Trial. Child Youth Care Forum.

[131] Kennard BD, Biernesser C, Wolfe KL, Foxwell AA, Craddock Lee SJ, Rial KV, Patel S, Cheng C, Goldstein T, McMakin D, Blastos B, Douaihy A, Zelazny J, Brent DA (2015). Developing a Brief Suicide Prevention Intervention and Mobile Phone Application: A Qualitative Report. Journal of Technology in Human Services.

[132] Tan Z, Liu X, Liu X, Cheng Q, Zhu T (2017). Designing Microblog Direct Messages to Engage Social Media Users With Suicide Ideation: Interview and Survey Study on Weibo. J Med Internet Res.

[133] LeMay R (2015). Student learning of violence prevention education concepts: a longitudinal analysis. Doctoral Dissertation.

[134] de Andrade NNG, Pawson D, Muriello D, Donahue L, Guadagno J (2018). Ethics and artificial intelligenceuicide prevention on Facebook. Philosophy & Technology.

[135] McKernan LC, Clayton EW, Walsh CG (2018). Protecting Life While Preserving Liberty: Ethical Recommendations for Suicide Prevention With Artificial Intelligence. Front. Psychiatry.

