# Supplementary material for: Information and Communication Technology Use in Suicide Prevention: Scoping Review
Source: J Med Internet Res. 2021 May 4;23(5):e25288. doi: 10.2196/25288 (PMC8132980; doi:10.2196/25288)
Supplement: Multimedia Appendix 1 [file jmir_v23i5e25288_app1.docx]

**Multimedia Appendix 1**

Search strategy syntax specific to each consulted database.

| Database | Syntax |
| --- | --- |
| PubMed | ((((suicid*[TIAB] OR "self harm"[TIAB] OR "self-injurious behavior"[MeSH Terms] OR "self-destructive behavior"[MeSH Terms] OR "suicidal ideation"[MeSH Terms] OR "attempted suicide"[MeSH Terms] OR "deliberate self harm"[MeSH Terms] OR "self- injurious behaviors"[MeSH Terms]))) AND ((((algorithm*[TIAB] OR facebook OR internet OR online OR twitter OR "big data" OR cyber* OR instagram OR snapchat OR periscope OR "ask.fm" OR internet OR "machine learning"[MeSH Terms] OR "deep learning"[TIAB] OR "machine learning"[TIAB] OR "algorithms"[MeSH Terms]))) OR (("mobile tech*"[TIAB] OR "social media"[TIAB] OR "electronic health record"[TIAB] OR "mining"[MeSH Terms] OR "mining"[TIAB] OR "neural network"[TIAB] OR "artificial intelligence"[MeSH Terms] OR "artificial intelligence"[TIAB] OR "computational intelligence"[TIAB] OR "natural language processing"[MeSH Terms] OR "natural language processing"[TIAB] OR "NLP"[TIAB] OR "mobile applications"[MeSH Terms] OR "cell phones"[MeSH Terms] OR "text messaging"[MeSH Terms])))) AND (((Intervention*[TIAB] OR postvention*[TIAB] OR (clinic*[TIAB] AND chang*[TIAB]) OR monitoring[TIAB] OR therapeut*[TIAB] OR management[TIAB] OR treatment*[TIAB])) OR ((“Program development”[MeSH Terms] OR “Program evaluation”[MeSH Terms] OR "Preventive Health Personnels"[MeSH Terms] OR "Safety Management"[MeSH Terms] OR “Occupational Health Services”[MeSH Terms] OR “Health Promotion”[MeSH Terms] OR “Patient Care Planning”[MeSH Terms] OR “Postvention”[MeSH Terms] OR “Treatment effectiveness evaluation”[MeSH Terms] OR “Pathology, Clinical”[MeSH Terms] OR “Clinical trial”[MeSH Terms] OR “Clinical decision making”[MeSH Terms] OR “Decision support systems, Clinical”[MeSH Terms] OR “Clinical Medicine” OR “Therapies, Investigational”[MeSH Terms] OR “Integrated Advanced Information Management Systems”[MeSH Terms] OR “Community Health Planning”[MeSH Terms] OR “Community Participation”[MeSH Terms] OR “Community Health Services”[MeSH Terms] OR “Delivery of Health Care”[MeSH Terms] OR “Community-Based Participatory Research”[MeSH Terms] OR “Psychotherapy”[MeSH Terms] OR “Early intervention”[MeSH Terms] OR “Crisis intervention”[MeSH Terms] OR “Clinical decision-making”[MeSH Terms] OR “Brief Therapy”[MeSH Terms] OR “Early intervention”[TIAB] OR “Treatment”[MeSH Terms] OR “Self-Help Techniques“[MeSH Terms] OR “Telemedicine”[MeSH Terms] OR “School-based intervention” OR “Workplace intervention”[TIAB] OR “Educational program”[TIAB] OR “Self-Help”[TIAB]))) |
| PsycInfo | (((((AnyField:(algorithm))) OR ((AnyField:("artificial intelligence"))) OR ((AnyField:("computational intelligence"))) OR ((AnyField:("big data"))) OR ((AnyField:("data mining"))) OR ((AnyField:("Pattern Discrimination"))) OR ((AnyField:("Pattern Recognition"))) OR ((AnyField:("Artificial Intelligence"))) OR ((AnyField:("Automated Information Processing"))) OR ((it:("Machine Learning"))) OR ((AnyField:("neural network"))) OR ((AnyField:("neural networks"))) OR ((AnyField:("Artificial Neural Networks"))) OR ((it:("Computational Modeling"))) OR (AnyField:(facebook)) OR (AnyField:(internet)) OR (AnyField:(online)) OR (AnyField:(twitter)) OR (AnyField:("big data")) OR (AnyField:(cyber*)) OR (AnyField:(instagram)) OR (AnyField:(snapchat)) OR (AnyField:(periscope)) OR (AnyField:("ask.fm")) OR (AnyField:(internet)) OR (AnyField:("deep learning"))) OR (((AnyField:(mobile)) AND (AnyField:(tech*))) OR (ti:("social media")) OR (abstract:("social media")) OR (ti:("electronic health record")) OR (abstract:("electronic health record")) OR (it:("Data mining")) OR (AnyField:("mining")) OR (AnyField:("neural network")) OR (AnyField:("neural networking")) OR (AnyField:("neural networks")) OR (AnyField:("neural networksbased")) OR (it:("artificial intelligence")) OR ((AnyField:(artificial)) AND (AnyField:(intelligence))) OR (AnyField:("artificial intelligence")) OR ((AnyField:(computational)) AND (AnyField:(intelligence))) OR (AnyField:("computational intelligence")) OR (it:("Computer Assisted language Learning")) OR ((AnyField:(natural)) AND (AnyField:(language)) AND (AnyField:(processing))) OR (AnyField:("natural language processing")) OR (it:("computer applications")) OR (it:("Cellular Phones")) OR (it:("text Messaging")))) AND ((abstract:(Intervention*) OR ti:(Intervention*)) OR (abstract:(clinic*) AND abstract:(chang*)) OR (ti:(clinic*) AND ti:(chang*)) OR (abstract:(program*) OR ti:(program*)) OR (abstract:(monitoring) OR ti:(monitoring)) OR (abstract:(therapeut*) OR ti:(therapeu*)) OR (abstract:(management) OR ti:(management)) OR (abstract:(treatment*) OR ti:(treatment)) OR (IndexTerms:(intervention) OR IndexTerms:(postvention) OR IndexTerms:("Clinical change") OR IndexTerms:(program) OR IndexTerms:(policies) OR IndexTerms:(policy) OR IndexTerms:(monitoring) OR IndexTerms:(therapeutics) OR IndexTerms:(management) OR IndexTerms:(treatment) OR IndexTerms:("Crisis Intervention") OR IndexTerms:("Early Intervention") OR IndexTerms:("School Based Intervention") OR IndexTerms:("Workplace Intervention") OR IndexTerms:("Crisis Intervention Services") OR IndexTerms:("Risk Management") OR IndexTerms:("Educational Programs") OR IndexTerms:("Government Programs") OR IndexTerms:("Response to Intervention") OR IndexTerms:("Adjunctive Treatment") OR IndexTerms:(Aftercare) OR IndexTerms:("behavior Modification") OR IndexTerms:("Computer Assisted Therapy") OR IndexTerms:("Cross Cultural Treatment") OR IndexTerms:("Health Care Services") OR IndexTerms:("Interdisciplinary Treatment Approach") OR IndexTerms:("Self-Help Techniques") OR IndexTerms:("Therapeutic Processes") OR IndexTerms:("Treatment Outcomes") OR IndexTerms:("Change Strategies") OR IndexTerms:("Community Services") OR IndexTerms:("Health Care Delivery") OR IndexTerms:("Health Care Policy") OR IndexTerms:("Mental Health Services") OR IndexTerms:("Telemedicine") OR IndexTerms:("Treatment Effectiveness Evaluation"))) AND ((IndexTerms:("Crisis Intervention Services") OR IndexTerms:("Self-Destructive Behavior") OR IndexTerms:("Self-Injurious Behavior") OR IndexTerms:("Suicidal Ideation") OR IndexTerms:("Suicide") OR IndexTerms:("Suicide Prevention") OR IndexTerms:("Suicidology") OR IndexTerms:("Attempted Suicide") OR IndexTerms:("Crisis Intervention Services")) OR (AnyField:(suicid*) OR AnyField:("suicide attempt"))) |
| Sociological Abstracts | (ti/ab((algorithm* OR facebook OR internet OR mining OR online OR twitter OR "big data" OR cyber* OR instagram OR snapchat OR periscope OR "Epidemiological Monitoring" OR "machine learning" OR "deep learning" OR "machine learning" OR "natural language processing" OR "data mining" OR "artificial intelligence" OR " mobile tech* " OR " social media " OR " electronic health record " OR " mining " OR "neural network*" OR "artificial intelligence " OR " computational intelligence " OR " natural language processing " OR " mobile applications " OR " cell phones " OR " text messaging")) AND ti/ab((suicid* OR "self harm" OR "self-injurious behavior" OR "self- destructive behavior" OR "suicidal ideation" OR "attempted suicide" OR "deliberate self harm" OR "self-injurious behaviors"))) AND (Intervention* OR postvention OR (clinic* AND chang*) OR program* OR monitoring OR therapeut* OR management OR treatment*) |
| IEEE Xplore | (suicid* OR "self harm" OR "self-injurious behavior" OR "self-destructive behavior" OR "suicidal ideation" OR "attempted suicide" OR "deliberate self harm" OR "self-injurious behaviors") |
